# Physical activity as a preventive measure against overweight, obesity, infections, allergies and cardiovascular disease risk factors in adolescents: AFINOS Study protocol

**DOI:** 10.1186/1471-2458-9-475

**Published:** 2009-12-19

**Authors:** Oscar L Veiga, Sonia Gómez-Martínez, David Martínez-Gómez, Ariel Villagra, María E Calle, Ascensión Marcos, AFINOS Study Group

**Affiliations:** 1 Department of Physical Education, Sport and Human Movement, Faculty of Teacher Training and Education, Universidad Autónoma de Madrid, Campus de Canto Blanco. Ctra de Colmenar Km 11. E-28049, Madrid, Spain; 2 Immunonutrition Research Group, Department of Metabolism and Nutrition, Instituto del Frío-ICTAN, Spanish National Research Council (CSIC), C/José Antonio Novais 10, E-28040 Madrid, Spain; 3 Department of Preventive Medicine and Public Health, Faculty of Medicine, Universidad Complutense de Madrid, Madrid, Spain. Ciudad Universitaria, S/N, E- 28040 Madrid, Spain

## Abstract

**Background:**

Prior studies addressing the impacts of regular physical activity or sedentary habits on the immune system have been conducted in adults and laboratory settings. Thus, it is practically unknown how a healthy active lifestyle could affect low-grade inflammation processes, infections or allergies in young persons. The AFINOS Study was designed to determine the relationship between the regular physical activity levels of adolescents and overweight, infection, and allergies along with the presence of metabolic and immunological biomarkers of a deteriorated health status. A further objective of the AFINOS Study is to assess the health status and lifestyle habits of an adolescent population in an effort to identify any protective factors that could be used as preventive measures, since many chronic diseases and their associated co-morbidities often persist from adolescence into adulthood.

**Methods/Design:**

This study was conducted as three separate sub-studies in three different populations as follows: (a) Study 1 was performed on a population sample of adolescents; (b) Study 2 on the adolescents' parents; and (c) Study 3 on a subset of the adolescents from Study 1. Study 1 assessed health and lifestyle indicators through a questionnaire administered to a representative sample of adolescents from the Madrid Region (n = 2400) aged 13 to 16 years. In Study 2, the parents of the teenagers participating in Study 1 were required to fill out a questionnaire. Finally in Study 3, body composition, physical activity, health-related physical fitness, and blood measurements were determined in a subset (n = 200) of the individuals included in Study 1.

**Discussion:**

This paper describes the rationale, design, and methodologies used in the AFINOS Study. This multidisciplinary, multicenter study seeks to evaluate several aspects of existing relationships between routine physical activity/sedentary behaviour and several health status markers, specifically those related to the immune system. The results of this cross-sectional study will serve for comparisons with the available data obtained in laboratory settings and in adults. In addition, knowledge regarding the health status and lifestyle habits of Spanish adolescents and their parents will be useful for designing preventive measures.

## Background

A link between physical activity and health has been inferred from the epidemiological evidence obtained over the past few decades. However, although this link has been clearly established in adults [1,2], knowledge about the impacts of physical activity on the health of children and adolescents is still lacking. To examine this issue, the US Centers for Disease Control conducted a review of the scientific literature that was published in 2005 [3]. This review concluded that moderate to vigorous physical activity in children and adolescents confers protection against a considerable number of health disorders, such as obesity or cardiovascular and metabolic diseases (high blood pressure, an altered blood lipid profile, metabolic syndrome, diabetes or inflammation). A further conclusion was that physical activity produces health benefits related to overall physical fitness (aerobic fitness, strength and muscular endurance) and bone density along with improved symptoms of asthma and several aspects of mental health (anxiety, depression, self-concept, academic performance, memory and classroom behaviour). On the other hand, the report highlights that there is insufficient evidence to clarify the potential effects of physical activity on the immune system of children and adolescents.

The relationship between physical activity and several health indicators in children and adolescents needs addressing further if we are to improve current knowledge of the effects physical activity may have on the health of individuals of this age. To date, the extent to which an active or inactive lifestyle could alter the immune system of adolescents remains unclear. Most studies that have assessed the relationship between physical activity and immunological markers have been carried out in laboratory settings, in which only information on the acute effects of isolated exercise sessions can be obtained. Moreover, most of these studies have been performed in adults [4] and very few investigations have examined the issue in children or adolescents [5]. According to the literature, light or moderate physical activity may enhance the immune system while intense physical activity could have the opposite effect of depleting the system [5]. There is therefore a need to assess under free-living conditions the effects on the immune system of different levels of physical activity. This type of investigation will provide insight into the long-term chronic effects of physical exercise on the health of adolescents, as an age group of particular interest, since any benefits or negative repercussions are likely to continue into adulthood.

Obesity is a health problem that has been declared by the WHO as a global epidemic, whose prevalence has been growing rapidly in many areas of the world [6]. Especially worrying, is the rapidly increasing incidence of overweight or obesity observed in the young people of many countries [7]. This trend indicates there will be a rise in the prevalence of obesity in adult populations of the near future accompanied by a wider spectrum of obesity-related diseases at increasingly earlier ages. Due to the high economic and social costs associated with obesity and its co-morbidities [8,9], obesity prevention is a priority objective that requires further knowledge of the factors that are converting the present generation of children and adolescents into the most obese in history. In Spain, the situation is similar to that of any other developed country, and we are seeing a rapidly rising trend in the number of children and adolescents of both sexes who are overweight or obese [10,11]. To combat this outbreak, the Spanish health authorities have recently put into practice a strategy targeted at the prevention of obesity and overweight [12].

There is a clear genetic genetic component that predisposes a person to overweight-obesity [13] and other health disorders [14]. Thus, knowing the family history of body weight status and immune system related disorders (infections and allergies) is extremely useful. In addition, the home environment will often determine the healthy or non-health lifestyle of an adolescent. Hence, information regarding lifestyle in the family environment of adolescents can improve our understanding of their health status and predisposition to disease [15].

Physical inactivity seems to be a prominent risk factor for developing overweight and obesity. Despite cross-sectional studies having only found moderate relationships between levels of physical activity and weight status, prospective studies have linked low levels of physical activity with high weight gain over time [16]. In addition, experimental studies have shown that physical exercise increases the efficacy of weight loss programmes and seems to be a critical factor for weight maintenance over time [17]. Secular trends over the last forty years have also revealed that increased patterns of overweight and obesity show a stronger association with intensely sedentary behaviour than with a high calorie intake [18,19]. Exercise programmes have been demonstrated to reduce the body fat percentage in overweight children and adolescents [3] and there is also evidence indicating that active children have lower body fat proportions than their inactive peers.

Physical activity can be linked to most biological cardiovascular risk factors and metabolic disorders [20]. Thus, physical activity has been shown to reduce blood pressure in adults with high blood pressure, and also to reduce blood triglyceride levels and increase HDL-c concentrations. Studies have also shown that physical activity may improve insulin resistance and glucose tolerance, along with coagulation characteristics linked to thromboembolic phenomena. Similar beneficial effects to those found in adults have been described in children and adolescents related to glucose regulation, insulin sensitivity, as well as high blood pressure, HLD-c and triglyceride levels [3].

Current research efforts are being made to address the existing relationship between obesity and immunocompetence [21] and its impact on the long-term development of metabolic disorders and cardiovascular diseases. Obese subjects suffer from sub-clinical inflammation, which has been suggested to induce insulin resistance and metabolic syndrome [22]. Obesity could also affect the defence capacity of the immune system making obese subjects more susceptible to infection [23] or allergies [24].

### Study aims

The main aim of the AFINOS Study (La Actividad Física como Agente Preventivo del Desarrollo de Sobrepeso, Obesidad, Alergias, Infecciones y Factores de Riesgo Cardiovascular en Adolescentes; Physical Activity as a Preventive Measure for Overweight, Obesity, Infection, Allergies and Cardiovascular Risk Factors in Adolescents) is to examine relationships between regular physical activity levels in adolescents and overweight, infection and allergies, along with metabolic and immune system markers used to evaluate their health status. Our working hypothesis is that adolescents who more often undertake physical activity (excluding intensive training targeted at sports performance) are healthier and enjoy a better quality of life. In addition, this study seeks to establish the general prevalence of overweight-obesity and immune system-related disorders (infections and allergies) in the study population (adolescents aged 13 to 16 living in the Madrid region). Also examined was the potential influence of the family environment and lifestyle in developing a disease or acquiring (un)healthy habits that could eventually lead to an obesity-related disorder.

The study includes three surveys conducted on three different study populations: (a) Study 1 performed on a sample of 2400 adolescents; (b) Study 2 on a sample comprised of the parents of these adolescents; and (c) Study 3 performed on 200 of the adolescents included in (a) to assess in detail their physical activity levels and health status. The aims of each of these sub-studies are outlined below:

(a) Aims of Study 1 conducted on a representative sample of adolescents living in the Madrid region

1. To determine the prevalences of overweight, obesity, infections and allergies.

2. To assess physical activity and sedentary habits.

3. To assess basic eating habits.

4. To assess the relationship between sedentary lifestyle and the prevalence of health disorders (obesity, infections and allergies).

5. To asses the relationship between sedentary lifestyle and academic performance.

(b) Aims of Study 2 conducted on the adolescents' parents

1. To assess physical activity and sedentary habits.

2. To assess basic eating habits.

3. To assess the relationship between sedentary lifestyle and the incidence of certain chronic diseases (obesity, infections, allergies, cardiovascular risk factors and metabolic or cardiovascular diseases).

(c) Combined aims of Studies 1 and 2

1. To assess the relationship between obesity and susceptibility to infection in the adolescents and their parents.

2. To assess the relationship between physical activity and physical inactivity patterns in the adolescents and their parents.

3. To assess the relationship between the basic eating habits of the parents and basic eating habits of the adolescents involved in the study.

(d) Aims of Study 3 conducted on a subset of 200 adolescents

1. To assess the relationship between physical activity habits, physical fitness level and biochemical and immunological markers.

2. To assess the relationship between body composition (underweight/overweight/obesity) and biochemical and immunological profiles.

3. To assess the relationship between basic eating habits and biochemical and immunological profiles.

4. To assess the relationship between biochemical/immunological profiles and allergies, infections and cardiovascular risk factors.

## Methods/Design

The AFINOS Study is a multidisciplinary, cross-sectional, multicenter study endorsed by the National Research & Development & Innovation Plan of the Spanish Ministry of Education and Science, under the Strategic Line of Sport and Physical Activity. The three research groups involved are each specialized in a specific area of knowledge: (a) the Immunonutrition Research Group, Department of Metabolism and Nutrition, Scientific National Research Council (CSIC); (b) the Epidemiology Group, Department of Preventive Medicine and Public Health, Universidad Complutense de Madrid; and (c) the Physical Activity and Health Group. Department of Physical Education, Sport and Human Motricity, Universidad Autónoma de Madrid.

### Research universe and study samples

The AFINOS Study takes as universe, the population of adolescents aged 13 to 16 of the Madrid region (central Spain) and their parents. The Madrid Region is one of the country's most densely populated areas. With an extension of 8028 km^2 ^representing a mere 1.5% of the geographic area of Spain, the Madrid province is home to 13.5% of the entire Spanish population. According to the 2006 census of the National Statistics Institute http://www.ine.es, in this year there were 228,535 adolescents aged 13 to 16 living in the Madrid region, representing 12.7% of the Spanish adolescent population of this age group. Of these adolescents, 104,725 lived in Madrid City, 100,610 in its suburbs and 23,200 in the villages of the region.

The sample size for Study 1 was calculated using the Schlesselmann formula [25] for known population sizes, taking 0.05 as the maximum permissible error (reliability of 95%). In the calculation, we used the estimated variance for obese plus overweight subjects in our population of 20% indicated by data from the AVENA national study [10].

The final sample size calculated at 1998 individuals was increased by 20% to compensate for possible dropouts or data losses to give a final sample size of 2400 adolescents of both sexes.

According to the geographic distributions of adolescents in the Madrid region: 46% of the sample (1058 subjects) was selected from Madrid City, 35% (805 subjects) from its suburbs and 19% (435 subjects) from its villages. A randomised cluster sample procedure was used to select the sample. The first level of sampling was the entire set of secondary schools of the Madrid region. Thus, of 25 schools selected, 10 schools were in the city, 8 in the suburbs, and 7 schools in villages. The second level of sampling was school classroom/grade (2nd, 3rd and 4th years of Compulsory Secondary Education and first year of Non-Compulsory Secondary Education, according to the Spanish education system). The sample for Study 2 was comprised of the parents of the adolescents selected for Study 1.

For the detailed assessment of physical activity, physical fitness and haematological, biochemical and immunological profiles, the minimum sample size calculated was 182 individuals, for an alpha error of 5% and power of the study of 20% [25,26]. Given the possibility of 10% data losses, the final sample size was 200 individuals. This sample was a non-random convenience sample because of the high level of cooperation needed on the part of the schools and adolescents to determine all the variables decided upon. The sample was selected from the adolescents attending two of the schools taking part in Study 1.

### Pilot Study

Prior to the main study, a pilot study was conducted between March and May 2007. One hundred adolescents of both sexes of similar age to those recruited for the main study were selected to check the feasibility, understanding and reliability of the questionnaires. Similarly, the feasibility of the planned timetable for acquiring body composition and physical fitness data in the convenience sample was verified. The pilot study also served to train evaluators and standardize planned protocols of data collection.

### Data Collection

Data collection for Study 1 was undertaken by the epidemiological research group in the selected classrooms. The adolescents were requested to complete a questionnaire, which took 60 to 90 minutes. At the end of this session, the researchers gave another questionnaire to each adolescent for both their mother and father to fill out (Study 2) to be handed in a week later.

**Figure 1:**
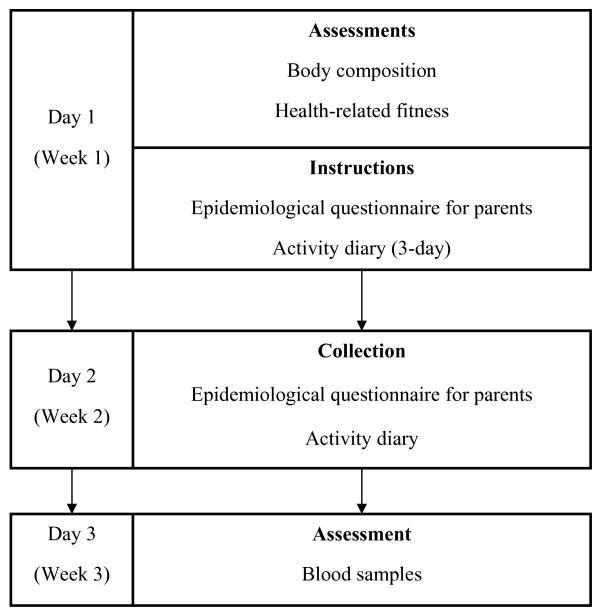
**Diagram showing the logistics of data collection for Study 3**.

For Study 3, data were collected over three consecutive weeks over the period November 2007 to February 2008. To recruit the adolescents, all the classes in one of the schools participating in the epidemiological study (Study 1) were invited to participate. Since we were unable to obtain a sample of 200 boys and girls from one school, a second school was invited to cooperate. Informative meetings were organized for the adolescents and their parents at the schools. At these meetings written information was provided detailing the project's objectives and how they would be contributing to the study. For the physical examination and biological sample collection, the adolescents and at least one of their parents or legal guardians signed an informed consent form. Figure 1 shows the data collection process in the convenience sample.

### Variables and study instruments

The questionnaire used for Study 1 assesses health status and was based on previous questionnaires used in several national and international health surveys conducted in adolescents [27-30]. This questionnaire was prepared by selecting the questions most suited to our research objectives. Additionally, some questions were designed by the researchers to obtain information on the prevalence of infections and allergies. This aspect has not been considered in any of the previous questionnaires. We also incorporated a specific questionnaire from the AVENA Study about food intake [31], as well as the SCOFF questionnaire [32] in its Spanish version [33] that assesses the risk of eating disorders, and 2 questionnaires designed to measure physical activity and sedentary habits: the Spanish adaptation of the Physical Activity Questionnaire for Adolescents [34], and an adaptation of the Sedentary Habits Questionnaire for Adolescents used in the HELENA Study [35]. To determine physical activity and food consumption habits during break time, two additional questionnaires designed by our research group were included: Recess Physical Activity Questionnaire (RPAQ) and Recess Food Intake Recall (RFIR). The main topics covered by the complete questionnaire are provided in Table 1.

**Table 1:** Categories assessed in the questionnaires

		Adolescents' questionnaire	Parents' questionnaire
Health Status	Chronic disabilities	√	√
	Back pain	√	√
	Infections	√	
	Allergies	√	
	Body mass index	√	√
	Eating disorders	√	√
	Medication	√	
	Dental care	√	
Lifestyle Indicators	Physical activity	√	√
	Physical activity beliefs and attitudes	√	√
	Sedentary habits	√	√
	Physical activity and sedentarism in school recess	√	
	Food intake	√	√
	Food intake in school recess	√	
	Dietary patterns	√	√
	Tobacco	√	√
	Alcohol	√	√
	Academic performance	√	
	Academic beliefs and attitudes	√	

Adolescents' questionnaire administered in Studies 1 and 3. Parents' questionnaire administered in Study 2.

For Study 2, information was gathered via a questionnaire specifically designed for the parents. Questions related to the incidence of infections or allergies in the adolescents were also included to assess the validity, via a multiple-informant process, of the replies provided by the adolescents and parents. In addition, parents were questioned about their own health status and lifestyle. Two questionnaires about physical activity and sedentary habits were incorporated into the parents' survey: the International Physical Activity Questionnaire (IPAQ) [36] and the sedentary habits questionnaire used in research on adolescents adapted for adults. The main health and lifestyle topics included in the final questionnaire administered to the parents are provided in Table 1.

In addition to the assessments undertaken in Studies 1 and 2, the health status of the subjects participating in Study 3 was assessed in detail. The following list provides the additional determinations made in this subset of 200 volunteers:

#### Body composition

Body mass index (BMI) was calculated as weight (kg) without shoes and with light-weight clothing, divided by height (m) squared. Weight was measured using a standardized scale ± 0.05 kg. Height was measured using a statiometer ± 1 mm incorporated into the scale. Skinfold and body circumference measurements were obtained according to the protocol used in the AVENA Study in which anthropometric measurements were made in over 2500 Spanish adolescents [37].

The following skinfold measurements were made on the left side of the body to the nearest 0.1 mm using a skinfold caliper (Caliper Holtain; Holtain Ltd., Wales, UK): (1) triceps, halfway between the acromion process and the olecranon process; (2) biceps, at the same level as the triceps skinfold, directly above the centre of the cubital fossa; (3) subscapular, about 20 mm below the tip of the scapula, at an angle of 45° to the lateral side of the body; (4) suprailiac, about 20 mm above the iliac crest and 20 mm towards the medial line; (5) thigh, at the midline of the anterior aspect of the thigh, midway between the inguinal crease and the proximal border of the patella; (6) calf, at the level of maximum calf circumference, on the medial aspect of the calf. Circumferences were measured using an nonextensible metric measuring tape (Seca 200; Seca, Hamburg, Germany) to the nearest 1 mm on the: (1) biceps; (2) contracted biceps; (3) waist; (4) hip; and (5) calf. Each set of anthropometric measurements was taken twice.

Bioelectrical impedance was determined using AKERN 101 equipment (AKERN Srl., Firenze, Italy). This method has been previously used in adolescents [38,39]. The AKERN 101 body impedance analyser uses a classical tetrapolar technique, whereby a constant sinusoidal current is injected at a fixed frequency of 50 KHz. The current level is kept constant at 800 microamperes on overall loads ranging from 1 to 4000 ohms. Subjects removed their socks, shoes and any metal jewellery before measurements.

Blood pressure was measured at the same time of the day using a validated digital automatic blood pressure monitor (OMRON M6, OMRON HEALTH CARE Co., Ltd., Kyoto, Japan) according to the International Protocol of the European Society of Hypertension [40]. Sexual maturity was assessed following the methodology described by Tanner and Whitehouse [41], which distinguishes 5 stages according to genital development and pubic hair in males, and breast development and pubic hair in females.

#### Physical activity and energy expenditure

To more precisely assess the physical activity and inactivity patterns of the subset of adolescents, besides completing the questionnaire, each subject was requested to keep a diary of energy expenditure and wear an ActiGraph GT1M accelerometer. This small lightweight uni-axial accelerometer has been widely validated for use in children and adolescents [42]. Following consensus recommendations [43], the subjects wore the accelerometer for 7 days. The level of activity was recorded in the instrument's memory every 15 seconds (15-s epoch) as recommended for this age group. Additionally, during the time the subjects wore the accelerometer, they completed the Bouchard 3-day activity diary [44]. The activity diary estimates the total daily energy expenditure of each subject on Thursday, Friday and Saturday. This diary divides each day into 96 15-min periods. The subject is instructed to think about the amount of energy expended during each 15-min period and record it using a scale of 1 to 9 according to the instructions in the diary.

#### Health-related fitness assessment

The tests selected for the evaluation of health related fitness in the AFINOS Study form part of the EUROFIT and FITNESGRAM batteries used to assess the components of physical fitness associated with health [45,46]. This methodology has been used in the AVENA and HELENA studies to assess adolescents aged between 13 and 16 from Spain and other European countries [47]. Below, we describe the specific tests performed.

##### Course-navette or 20-m shuttle run test

This test is probably the most frequently used to assess cardio-respiratory capacity in children and adolescents [48]. The subject runs a distance of 20 metres without interruption according to the pace marked by the test protocol. The pace of the test is ramped such that running speed increases by 0.5 km/h each minute or period. The test is finished when the subject is unable to complete the 20 metre distance within the specified time period marked by a loud signal. The last completed run indicates the final result of the test.

##### Handgrip strength

Using a handheld dynamometer (TKK 5101; Takei, Tokyo, Japan) the maximum isometric force that the musculature of the hand and arm can generate was determined. The subjects were allowed 2 attempts per hand in a standing position with the arm completely extended without touching any part of the body. The optimum grip of the dynamometer in adolescents is adjusted according to the size of the hand and the sex of the subject [49].

##### Flexed arm hang

This test measures the amount of time that the subject can maintain the maximum voluntary contraction while hanging from a bar (palms outward). The arms, shoulders and dorsal musculature force should remain with the body in the "up" position for as long as possible.

##### Trunk lift

The strength and flexibility of the trunk extension is measured by having the subject lie face down on a mat, elevating the upper portion of his body using the back muscles. This position is maintained while the distance between the chin and floor is measured.

##### Sit-up

This test included in the EUROFIT battery, measures the abdominal strength of each subject. The participants lie face up with their hips and knees at a 90° angle. The ankles rest on a chair and the arms are crossed over the chest with each hand placed on the opposite shoulder. From this position the subject contracts the abdominal muscles and sits up to touch his knees with his elbows. The number of repeated abdominal contractions accomplished in one minute is measured.

##### Bosco jumps

To evaluate the strength of the lower half of the body, the protocol proposed by Bosco [50] for use on the Bosco System force platform was used (ERGOJUMP-Plus BOSCO SYSTEM^®^; Byomedic, S.C.P., Barcelona, Spain). Three kinds of vertical jumps are performed: squat jump, countermovement jump, and abalakov. The subject performs the three types of jump on the platform and the Bosco System calculates the height of the jump from the amount of time spent in the air during the jump. In addition to determining the explosive strength of the lower extremities, the results obtained from the three jumps allow assessment of the coordination index and elasticity index.

##### Standing broad jump

Standing with feet together, the subject jumps as far as possible from a horizontal line. The score equals the distance between the starting line for the jump and the subject's heels.

##### 4 × 10-m shuttle run

Adapted from the 5 × 10-m included in the EUROFIT battery, the objective of this test is to assess motion velocity, agility and coordination. The subject runs 10 meters back and forth 4 times, as fast as possible. In each 10 metre run, the subject picks up a sponge, then runs back 10 metres, drops the sponge and picks up another sponge, repeating the same actions two more times, using a total of three sponges during the test (for the first 10 m run the subject does not carry a sponge).

##### Back saver sit and reach

this test included in the FITNESSGRAM battery measures the flexibility of the subjects using a standard size box. With one foot up against the front side of the box and the other leg flexed, the subjects must stretch the longest distance possible from a seated position. The back-saver sit and reach assesses the flexibility of each leg independently, whereas the traditional sit and reach assesses the flexibility of both legs together.

#### Haematological and biochemical profiles

A fasting blood sample was obtained from the cubital vein in the early morning at the schools attended by the adolescents in the subset. 16 ml of blood was drawn from each subject and aliquoted into: 1 tube containing EDTA (4.5 ml), 1 tube containing EDTA (1.5 ml), and 1 tube containing dry gel for serum (10 ml). Given the labile nature of some of the molecules to be analyzed, the tube containing 1.5 ml of anticoagulated blood with EDTA is processed *in situ *with aprotinin as protease inhibitor [51]. Once the blood was extracted, it was immediately transported to the Instituto del Frio where the 4.5 ml of anticoagulated blood in EDTA was aliquoted into 2 parts and one sent to an outside laboratory to obtain haemogram data and the other one immediately analyzed in the CSIC laboratory to determine lymphocyte subpopulations. The remainder of the blood (EDTA treated and dried gel) was centrifuged, plasma and serum removed and then frozen at -80°C to be analyzed later.

The haematological study (haemogram) was performed using an automatic cell counter (ABX 120DX Horiba, Spain). The variables obtained were: (1) red blood cell, white blood cell, neutrophil, lymphocyte, monocyte, basophil, eosinophil and platelet counts and leukocyte profile; and (2) haemoglobin concentration, haematocrit and the haematic indices: mean corpuscular volume (MCV), mean corpuscular haemoglobin (MCH), concentration of mean corpuscular haemoglobin (CMCH), and red cell distribution width (RDW).

The biochemical profile included: 1) the plasma lipid triglycerides, total cholesterol, HDL-c, LDL-c (by enzymatic colorimetric methods) and apolipoproteins B and A1 (by turbidometry); and 2) the metabolic regulators glucose, total proteins, iron, urea and uric acid (by enzymatic colorimetric methods), ferritin and pre-albumin (by turbidometry). All determinations were analysed in serum by using an AU2700 Olympus analyser.

#### Immunological profile

A novel feature of this study was the inclusion of immunological variables based on the results of previous studies such as AVENA and HELENA [31,35]. The following determinations were made with the objective of maximizing the available resources.

##### Cell-mediated immune system

(1) the lymphocyte subsets CD3+ (mature T lymphocytes), CD4+ (T helper lymphocytes), CD8+ (T cytotoxic lymphocytes), CD16+/56+ (natural killer cells), CD45RA+ and CD45RO+ (native and memory T cells, respectively) determined by flow cytometry according to Baker [52]; (2) "in vivo" cell-mediated immune function through the analysis of serum cytokines: IL-2, IL-4, IL-5, IL-10, TNF-α, IFN-γ, IL-8, IL-1β, IL-6) by flow cytometry and TGF-β by Enzyme-Linked ImmunoSorbent Assay, ELISA

##### Humoral immune system

(1) the lymphocyte subset CD19+ (B lymphocytes) by flow cytometry according to Baker [52]; (2) humoral immune function assessed via serum immunoglobulins (Ig G, A, M) by turbidimetry using the AU 2700 (Olympus) and Ig E measured using the chemoluminescence instrument ADVIA CENTAUR (Siemens).

##### Innate immune system

serum complement factors C3 and C4 by turbidimetry (AU 2700) (Olympus); and (2) acute phase proteins: serum C-reactive protein by turbidimetry (AU 2700) (Olympus), and serum caeruloplasmin levels measured by nephelometer (Nephelometer Behring) (Dade-Behring).

##### Hormones and adhesion molecules

(1) the serum cell adhesion molecules sE-selectin, sICAM-1, sVCAM-1 by flow cytometry, sL-selectin by ELISA; (2) adipocytokines in serum via adiponectin by flow cytometry; (3) the gut hormones ghrelin, insulin, leptin, peptide YY in plasma treated with aprotinin analyzed by flow cytometry; and (4) the pituitary hormones thyroid stimulating hormone (TSH), luteinising hormone (LH), follicle-stimulating hormone (FSH), growth hormone (GH), prolactin, adenocorticotropic hormone (ACTH) by flow cytometry.

#### Ethical aspects

The study was conducted according to the ethical standards established in the 1961 Declaration of Helsinki (as revised in Hong Kong in 1989 and in Edinburgh, Scotland, in 2000). The AFINOS Study was approved by the Ethics Committee of the Puerta de Hierro Hospital (Madrid, Spain) and the Bioethics Committee of the Spanish National Research Council. Before participating in this study, all adolescents were informed of the nature of the study and gave their consent; parents also provided written consent. The data collected as well as any documents generated were numerically coded for anonymity and protection from unauthorized use by people not involved in the study. The information gathered in this study is strictly confidential. However, the participants and their parents were informed that the information could be accessed by the Spanish Health Authorities under the Law of Data Protection (LOPD 15/1999) covered by Spanish Law.

#### Statistical analysis

The final data will be analyzed using SPSS and SAS software. Frequencies and distributions of the variables will be determined using descriptive statistics that will serve to determine the general situation of the study subjects. Quantitative variables will be expressed as means and standard deviations of the mean, except for age, which will be expressed as year-by-year frequencies (13-14-15 and 16), and qualitative variables will be expressed as absolute frequencies of every category and percentages related to the total number of valid cases. The normal distribution of quantitative variables will be checked through adequate tests. To determine possible associations, the Chi-squared test will be used for qualitative observations and the student *t-*test to compare independent means of the quantitative data [26]. Pearson's correlation coefficient will be used to analyse two quantitative variables and if these are not verified as normally distributed the Spearman correlation will be used [53]. When analysis of several variables is needed, a multivariate analysis using the forward stepwise multiple regression method will be performed to construct models to explain the variability observed in the population and assess the combined effect of different variables. Risk profiles will be generated by multiple logistic regression, determining adjusted regression coefficients to calculate the odds ratio for each variable.

## Discussion

The AFINOS Study is a multidisciplinary, multicentre study designed to tackle in adolescents multiple aspects related to the relationship between the level of daily physical activity and several health markers, specifically those related to the immune system, cardiovascular risk factors or being overweight or obese. The main characteristic of this study is its dual approach in that it examines a population sample of adolescents and a convenience sample of this population, which is subjected to a meticulous analysis of levels of physical activity, physical fitness and health status, including an extensive study of immunological variables. A further innovative feature is the collection of additional information about the adolescents' families, which will be used to find out how the family environment could affect the health status of the adolescents.

The main contributions and strengths of the study are as follows. From the population study, extensive information on health markers and healthy habits can be obtained via questionnaires, allowing for a good description of the current health conditions of adolescents from the Madrid region. Especially relevant is the evaluation of sedentarism and physical activity, which was assessed in the population sample through different groups of items within the general questionnaire and two specific detailed questionnaires. The capacity to evaluate these variables in the adolescent population of Spain will be established by comparing the data obtained in the questionnaires with the information supplied by the accelerometer and diary in the convenience sample. This will provide detailed patterns of physical activity for the adolescents of the Madrid region, and also give an idea of the relative risks associated with sedentarism of developing the health problems for which information was collected. Additionally, the information on academic performance will allow for assessment of possible relationships of regular physical activity with this factor. This issue is of interest for discussions related to implementing daily physical education programmes in schools [54]. The information gained on the adolescents' family environment will help assess possible effects on acquired health habits in the adolescents themselves, as well as the appearance of specific health problems. Additionally, if there is a sufficiently high response rate, it may be possible to estimate the prevalences of physical activity habits, sedentarism, and of specific health habits in the adult population of the Madrid region.

The major strength of the convenience study is its detailed assessment of the patterns of physical activity and sedentary behaviour in the sample examined based on the concurrent use of objective (accelerometry) and subjective (diaries and questionnaires) methods. This will allow for determining the validity of the different subjective assessment methods for use in the Spanish adolescent population. Until now, very few instruments had been validated to assess physical activity and sedentarism in Spanish children and adolescents. Consequently, one of the expected applications of the study's results is to increase the number of validated tools available for future use in epidemiological and clinical studies in Spanish adolescents. Another major strength of the study consists of the exhaustive assessment of immunological profiles, including an extensive set of immunologic variables, which to the best of our knowledge have not been determined in similar studies performed in Spain or Europe. Additionally, the study fairly exhaustively examines health related fitness, including body composition variables and at-rest blood pressures.

One of the principal characteristics of this study is that it could be useful to assess relationships between physical activity and the immune system in free-living conditions targeted at gaining information on possible long-term adaptations that could be produced by regular physical activity in this population. In contrast, prior studies have mainly focused on the acute effects of exercise on the immune system under laboratory conditions. Moreover, the study's design will provide data on the possible contributions of different factors (overweight, obesity, physical activity/sedentarism and physical fitness) to the immunological status of adolescents. These data may also serve to highlight the influence of these factors on metabolic disorders (dyslipidemias, glucose intolerance and metabolic syndrome). Further, we should be able to glean information on physical activity and eating habits during school recess, which as far as we aware is a topic that has barely been touched. To obtain this type of information, two specific questionnaires have been created to help describe the habits of the adolescent population of Madrid in these specific areas.

The study has several limitations. Self reporting is one of the inherent problems affecting the quality of the information obtained in any inquiry through interviews and questionnaires. Infections and allergic processes are subject to seasonal change such that gathering information at one specific point in time will not precisely reflect the prevalence and incidence rates of these disorders. Another possible limitation has to do with the response rates of the parents of the adolescents in the survey process. A reduced response rate could produce an undetermined bias in the responses, which could influence the conclusions drawn from the results.

The limitations of Study 3 are the restricted sample size (n = 200), which is the consequence of the high costs of the immunological study (approximately 600€/subject) and the labour intense data collection process involved in measuring physical activity using the accelerometer. This sample size does not allow for extrapolating results to the population group under study. However, it is sufficiently large to establish relationships among the variables determined. An additional limitation is the non-random selection of the components of the convenience sample, which could introduce a bias associated with the specific characteristics of the school populations of the centres selected.

## Conclusions

In summary, the AFINOS Study seeks to gain insight into the lifestyle and health status of Spanish adolescents aged 13 to 16 years who live in the city of Madrid and its province. The study focuses on describing several health problems associated with the immunological status of the population (infections and allergies). In this way, it tries to evaluate the existing relationship between being overweight and habits of physical activity-sedentarism and the incidence of infections and allergies. Our exhaustive evaluation of the immunological and metabolic profiles of 200 adolescents will help us understand the existing link between physical activity habits or fitness levels and immunocompetence or metabolic health status. The results obtained in the convenience sample of this cross-sectional study will serve to undertake comparisons with the results of previous laboratory studies which seem to indicate that moderate exercise stimulates certain aspects of the immune response while intense exercise has undesirable effects [5]. In addition, the results obtained will serve to confirm previously reported findings of research into relationships between physical activity and being overweight or having a metabolic disorder in this age group [3].

## List of abbreviations

AVENA: Alimentación y Valoración del Estado Nutricional en Adolescentes [Food and Assessment of the Nutritional Status of Spanish Adolescents]; HELENA: Healthy Lifestyle in Europe by Nutrition in Adolescence; EDTA: ethylenediaminetetraacetic acid; HDL-c: high density lipoprotein cholesterol; LDL-c: low density lipoprotein cholesterol; SAS: Statistical Analysis Software; SPSS: Statistical Package for the Social Sciences; WHO: World Health Organization.

## Competing interests

The authors declare that they have no competing interests.

## Authors' contributions

All the authors contributed to the conception and design of the AFINOS Study and to drafting the manuscript. Final approval of the version to be published was obtained from each of the authors.

## Pre-publication history

The pre-publication history for this paper can be accessed here:

http://www.biomedcentral.com/1471-2458/9/475/prepub
